# First person – Meghan Barrett and Katherine Fiocca

**DOI:** 10.1242/bio.050187

**Published:** 2020-01-02

## Abstract

First Person is a series of interviews with the first authors of a selection of papers published in Biology Open, helping early-career researchers promote themselves alongside their papers. Meghan Barrett and Katherine Fiocca are co-first authors on ‘Larval mannitol diets increase mortality, prolong development, and decrease adult body sizes in fruit flies (*Drosophila melanogaster)*’, published in BiO. Meghan is a PhD candidate in the lab of Dr Sean O'Donnell at Drexel University, Philadelphia, USA, investigating the relationships between an organism's behaviour and morphology. Katherine is also a PhD candidate in the lab of Dr Sean O'Donnell, and is investigating how nutrition affects behaviour in social insects.


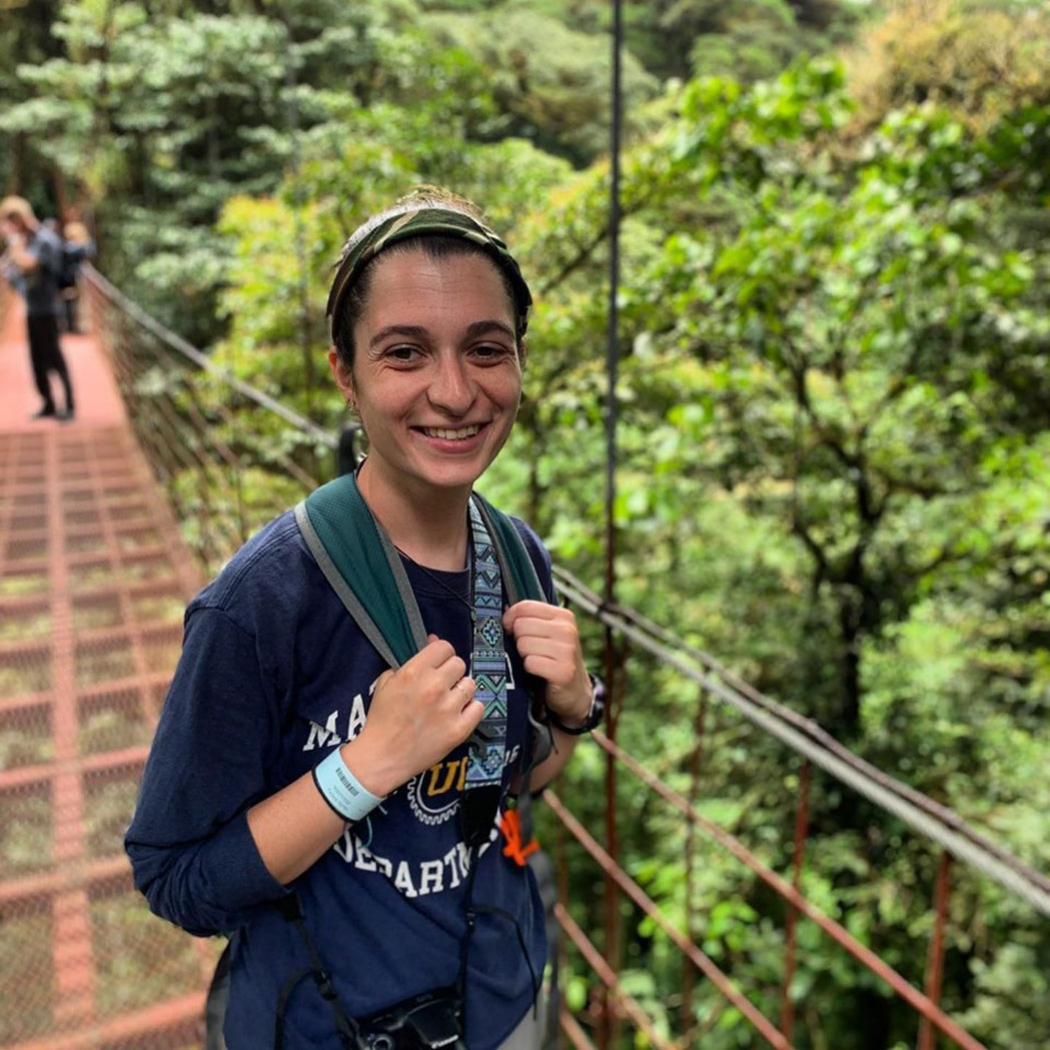


**Katherine Fiocca**

**What is your scientific background and the general focus of your lab?**

**M.B.:** I earned my BSc in 2016 from the State University of New York at Geneseo in Biology and English/Creative Writing and worked in Dr Jennifer Apple's lab, studying native bee diversity across a gradient of land management and intracolony relatedness in *Formica pergandei* ants. My thesis work in the O'Donnell lab focuses on morphological differentiation in the *Centris pallida* bee alternative reproductive tactic system, as well as the impact of different microclimates on bee behaviour.

**K.F.:** I earned my BSc in 2016 from Ursinus College in Biology and worked in a research lab run by Dr Mark Ellison of the Chemistry department, testing the use of nano-graphene as a vehicle for antibiotics and their effect on resistant *Escherichia coli*. Currently, I study a social paper wasp in Costa Rica, working to understand how nutrition affects reproduction potential and social behaviour among nestmates as part of my PhD studies under Sean O'Donnell at Drexel University.


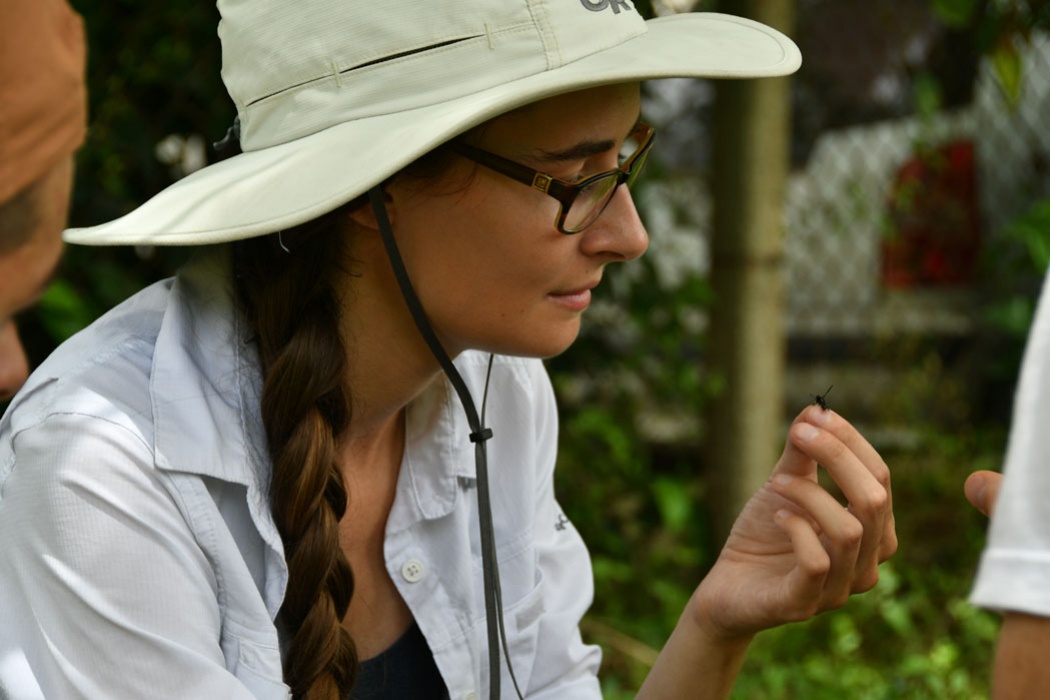


**Meghan Barrett**

**How would you explain the main findings of your paper to non-scientific family and friends?**

**K.F. & M.B.:** Fruit flies take longer to grow and are smaller when fed mannitol, an artificial sweetener, as larvae. These results are similar to the effects of high-sugar diets that make larvae insulin-resistant (a pre-cursor condition to type II diabetes in humans) and

“These results [...] demonstrate the important role of nutrition in regulating health and development.”

**What are the potential implications of these results for your field of research?**

**K.F. & M.B.:** Understanding the intricate relationships governing body size and developmental duration is a complex problem and we still know relatively little about what developmental variables impact this process. This work has added evidence to the importance of nutrition in regulating the body size/duration relationship and also shows that adult and developing insects may be impacted very differently by the same compounds.

**What has surprised you the most while conducting your research?**

**M.B.:** I really enjoyed how important observations came to this work – something I am used to in the field, but not so much in the lab. Katie and I were counting the larvae in the mannitol plates day after day and at some point we just said ‘Wow, these 0.8 M larvae are still so small compared to the control plates’ – and all the body size work came from just that casual observation.

**K.F.:** I was surprised to have been excited to work on an insect housed in the laboratory versus an insect out in the field. I realized the questions we were asking were similar to those that I am excited about for my thesis work, and that *Drosophila* offered a great model system for answering these kinds of questions.

**What changes do you think could improve the professional lives of early-career scientists?**

**M.B.:** The current scientific incentive structure for early-career researchers is set up to value short-term research productivity over all else – teaching, mentorship, scientific community building, equity initiatives, even thoughtful but long-term, high-quality research projects. Academia, and particularly tenured faculty and administrators, must resist the commodification of the professoriate by resetting incentive structures and fighting for improvements to the job market: better pay, more tenured positions, a reduction to the overworked and overabundant graduate student and adjunct labour pools, and a greater focus on quality work of all types over simple ‘science production’. We must incentivize what really matters: the educational experience and the value of learning, and this will benefit *all* stages of researchers (but especially early-career scientists and students).

**Figure:**
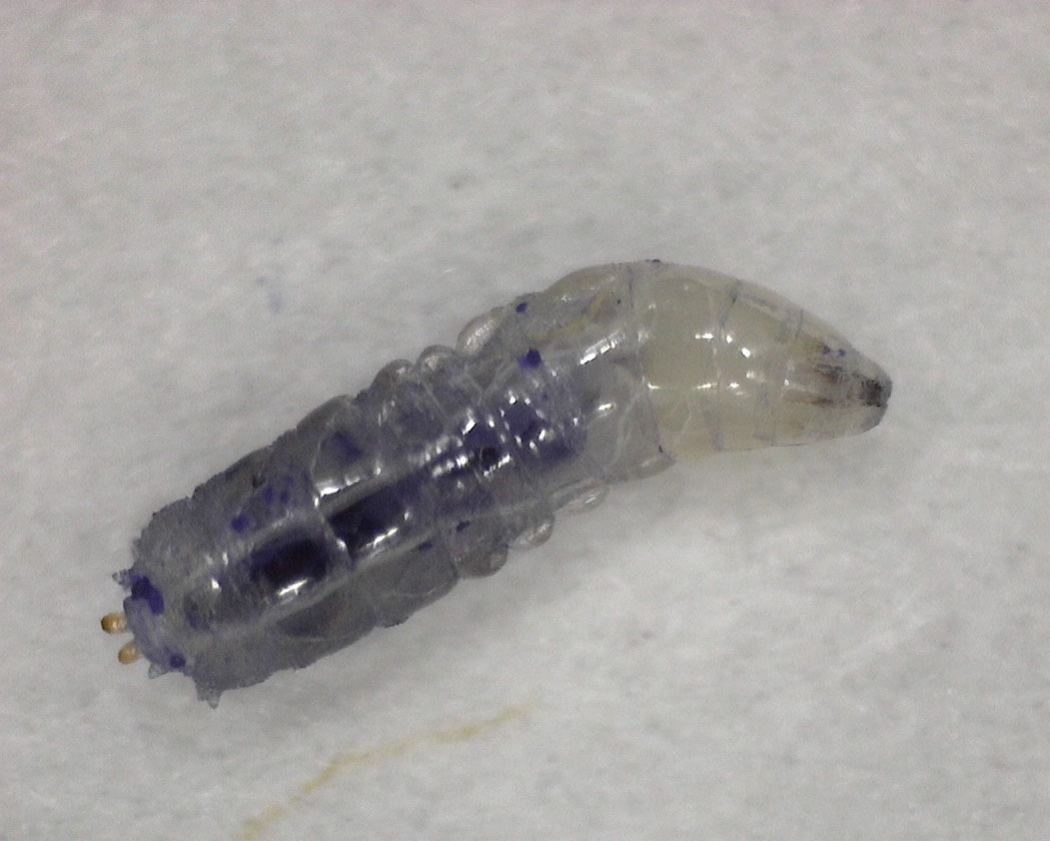
**A *D. melanogaster* larvae containing blue mannitol foods.**

“We must incentivize what really matters: the educational experience and the value of learning...”

**K.F.:** I think support from established colleagues is imperative to our success as early-career researchers. Whether that is financial support, research support, or emotional support, the path to success is more manageable when you have a support system, especially made up of those who have experienced and overcome similar challenges in academic science. Mentorship is an invaluable gift, but also requires conscience reflection and evaluation, and should be a skill practiced by researchers at all career stages.

**What's next for you?**

**M.B.:** ‘Next’ is so relative! Short-term, I'll be continuing my thesis work on sensory differentiation in alternative reproductive tactic systems while engaging in active-learning strategies and quality mentorship at Drexel. I also enjoy outreach and service, and hope to keep building my portfolio bringing science literacy (from process to results) to the people.

**K.F.:** I will be returning to Monteverde, Costa Rica to continue data collection on social paper wasps for my thesis work at Drexel University. I will be manipulating access to specific diets and tracking changes in social behaviour of *Mischocyttarus pallidipectus* paper wasps. Additionally, I will have the opportunity to resume my mentorship of Drexel undergraduates in the field, which is something that I really enjoy and look forward to every year.
